# Malignant glaucoma following gonioscopy-assisted transluminal trabeculotomy: a case report

**DOI:** 10.1186/s12886-022-02276-3

**Published:** 2022-02-08

**Authors:** Elena Bolletta, Danilo Iannetta, Antonio Moramarco, Luigi Fontana

**Affiliations:** 1 Ocular Immunology Unit, Azienda USL-IRCCS di Reggio Emilia, Viale Risorgimento 80, Reggio Emilia, Italy; 2 Ophthalmology Unit, DIMES, Alma Mater Studiorum, University of Bologna and S. Orsola-Malpighi Teaching Hospital, 40138 Bologna, Italy

**Keywords:** Gonioscopy-assisted transluminal trabeculotomy, Malignant glaucoma, Glaucoma surgery complication, Case report

## Abstract

**Background:**

To report a case of malignant glaucoma that developed after gonioscopy-assisted transluminal trabeculotomy (GATT).

**Case presentation:**

An 85-year-old male pseudophakic patient affected by pseudoexfoliative glaucoma (PEXG), unresponsive to medical glaucoma treatment, underwent uneventful GATT surgery. On the first day after surgery, the eye showed a shallow central and peripheral anterior chamber (AC) with a raised intraocular pressure (IOP) measured at 55 mmHg. Optical coherence tomography and ultrasound biomicroscopy confirmed the diagnosis of malignant glaucoma.

Laser iridotomy, posterior capsulotomy and hyaloidotomy were performed, and the patient was treated with atropine sulphate 1%, maximum topical and systemic ocular hypotensive drugs with no improvement in the IOP. Subsequently, the patient underwent pars plana anterior vitrectomy, resulting in deepening of the AC with opening of the iridocorneal angle and decrease of the IOP. No further postoperative complications were recorded, and the IOP remained controlled 12 months after surgery without antiglaucoma medications.

**Conclusions:**

Despite the minimally invasive profile of GATT, malignant glaucoma may develop after this procedure. Early recognition and prompt treatment are mandatory for preventing permanent visual loss.

## Background

Glaucoma is a progressive optic neuropathy that represents the second most prevalent cause of blindness worldwide [1]. The primary treatment modality in glaucomatous patients is medical therapy which aims to reduce aqueous production and/or to enhance aqueous outflow [2]. Despite medical treatment, in cases of inadequate IOP control, filtration surgery by trabeculectomy (TRAB) is considered the gold standard surgical procedure [3]. Although its high efficacy in reducing the IOP, TRAB is subject to several sight-threatening complications, including hypotony, choroidal effusion, suprachoroidal haemorrhage, endophthalmitis, and loss of light perception [4]. The use of minimally invasive glaucoma surgery (MIGS) has increased in the last few years thanks to its high safety profile, conjunctival sparing *ab interno* approach, absence of a filtering bleb, and rapid recovery after surgery. The IOP-lowering effect is achieved with a broad spectrum of mechanisms of action, a trabecular meshwork outflow enhancement and an increase in uveoscleral or subconjunctival drainage, with or without device implantation [5].

Gonioscopy-assisted transluminal trabeculotomy (GATT) is a minimally invasive surgical procedure introduced by Grover et al. in 2014 to manage open-angle glaucoma [6].

GATT has been shown to be safe and effective for treating adult primary open-angle glaucoma (POAG), primary congenital and juvenile glaucoma, and various forms of secondary glaucoma, including pigmentary, traumatic, steroid-induced, uveitic, and pseudoexfoliative [7].

Following GATT, the most common complication is transient hyphema, which can occur in up to 100% of patients within the first weeks after surgery [8, 9]. Other complications may occur more rarely, including Descemet’s membrane detachment, cyclodialysis cleft, suprachoroidal haemorrhage, vitreous haemorrhage, microcystic corneal edema, postoperative transient intraocular pressure (IOP) spikes, supraciliary effusion and cystoid macular edema [8, 10, 11].

Herein, we describe a previously unreported case of malignant glaucoma following GATT surgery and its management.

## Case presentation

An 85-year-old male Caucasian with pseudo-exfoliative glaucoma (PEXG), defined as an abnormal accumulation of fibrillo-granular protein (“pseudoexfoliation material”) produced in the eye [12], was referred to our ophthalmology unit (Azienda USL – IRCCS di Reggio Emilia, Reggio Emilia, Italy) with a raised IOP on maximum topical treatment. Four months earlier, the patient had undergone cataract surgery in another hospital. According to the available medical records, phacoemulsification surgery was uneventful, with the implantation of a single-piece intraocular lens (IOL) in the capsular bag. Preoperatively, the axial length was 23.46 mm, the anterior chamber depth was 3.52 mm, and the patient was not hyperopic.

When we first examined the patient, his visual acuity was 20/20 (Snellen) unaided, and the IOP, measured with Goldmann applanation tonometry (Haag-Streit, Koeniz, Switzerland), was 25 mmHg despite three glaucoma drugs. Anterior segment examination showed a clear cornea, a deep anterior chamber, and a well-centred IOL in the posterior chamber. Typical pigment loss from the pupil margin was present (“moth eaten pupil”). Gonioscopy using a Goldmann three-mirror lens revealed an open angle with no synechiae and Sampaolesi’s line, defined as pigment deposition anterior to Schwalbe’s line, in all quadrants. Dilated fundoscopy showed optic disc cupping and neuroretinal rim thinning.

**Fig. 1:**
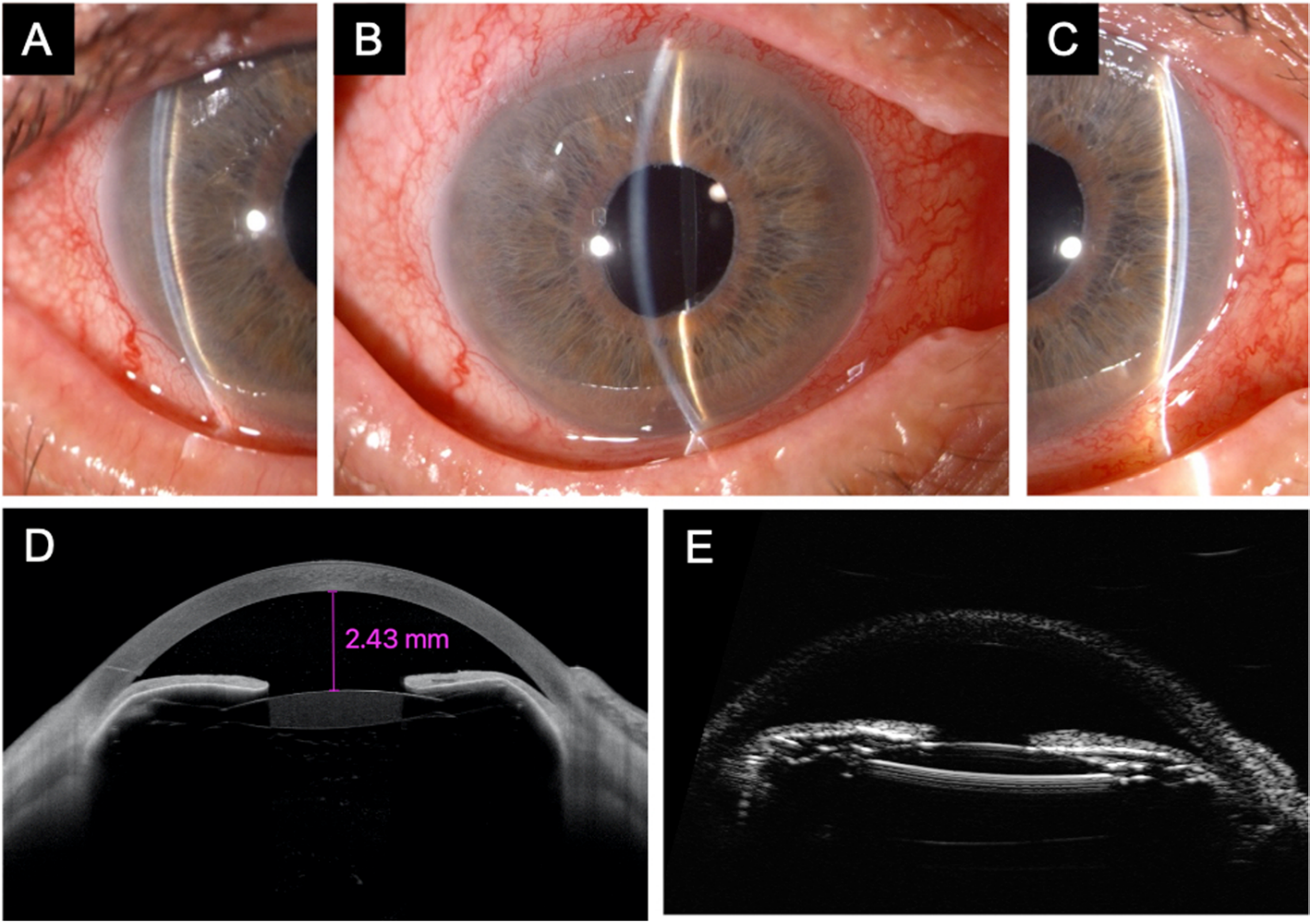
Anterior segment slit lamp biomicroscopy of the right eye 1 day after gonioscopy-assisted transluminal trabeculotomy. The central and peripheral anterior chambers (**A**, **B**, and **C**) are shallow (Van Herick grade 1). Anterior segment optical coherence tomography (**D**) and ultrasound biomicroscopy (**E**) show reduced central and peripheral anterior chamber depth, closed angle, and anterior displacement of the iris-lens diaphragm. The intraocular pressure was 55 mmHg, and a diagnosis of malignant glaucoma was made

Thus, GATT was planned using the technique described by Grover et al. [6]. Briefly, a 2.2-mm limbal incision was created at the clear temporal cornea. The surgeon visualised the nasal angle using a Swan–Jacob goniolens with proper orientation of the microscope and the patient’s head. The anterior chamber was filled with a cohesive viscoelastic material (Healon GV® Pro, Johnson & Johnson Vision, New Brunswick, NJ, USA). A 1- to 2-mm goniotomy was performed at the nasal angle using a 20-gauge sclerotome. The distal tip of an illuminated microcatheter (iTRACK, Ellex iScience Inc., Fremont, CA, USA) was inserted into the Schlemm’s canal at the goniotomy site and advanced circumferentially. Once the distal tip of the catheter circumnavigated the canal for 360°, the catheter was retrieved and externalised, creating an *ab interno* complete trabeculotomy. The viscoelastic material was rinsed, and the anterior chamber was half-filled with a dispersive viscoelastic substance (Eyefill, Bausch & Lomb, Bridgewater, NJ, USA) to tamponade bleeding from the angle. No intraoperative complications were recorded. One day after surgery, the patient presented ocular pain, blurred visual acuity reduced to counting fingers, and an IOP of 55 mmHg, as measured by Goldmann applanation tonometry (Haag-Streit, Koeniz, Switzerland). During slit-lamp examination, peripheral and central anterior chamber (AC) depth were reduced (Van Herick grade 1; Fig. 1A, B, and C) with a closed angle at gonioscopy (Shaffer grade 0). Anterior segment optical coherence tomography (AS-OCT) (SL-OCT; Heidelberg Engineering, Heidelberg, Germany) and ultrasound biomicroscopy (UBM) revealed anterior displacement of the iris-lens diaphragm (Fig. 1D and E) with a shallow anterior chamber of 2.43 mm. Posterior-segment B-scan ultrasonography showed no vitreous or choroidal abnormalities. According to the UBM description of Trope et al. [13], a diagnosis of pseudophakic malignant glaucoma was made.

The patient was initially treated with 250 ml of 20% mannitol intravenous infusion, oral acetazolamide, topical beta-blockers, alpha agonists, and cycloplegics. On the same day, peripheral neodymium-doped yttrium aluminium garnet (Nd: YAG) laser iridotomy, central posterior capsulotomy, and hyaloidotomy were performed with no resolution of symptoms and signs.

**Fig. 2:**
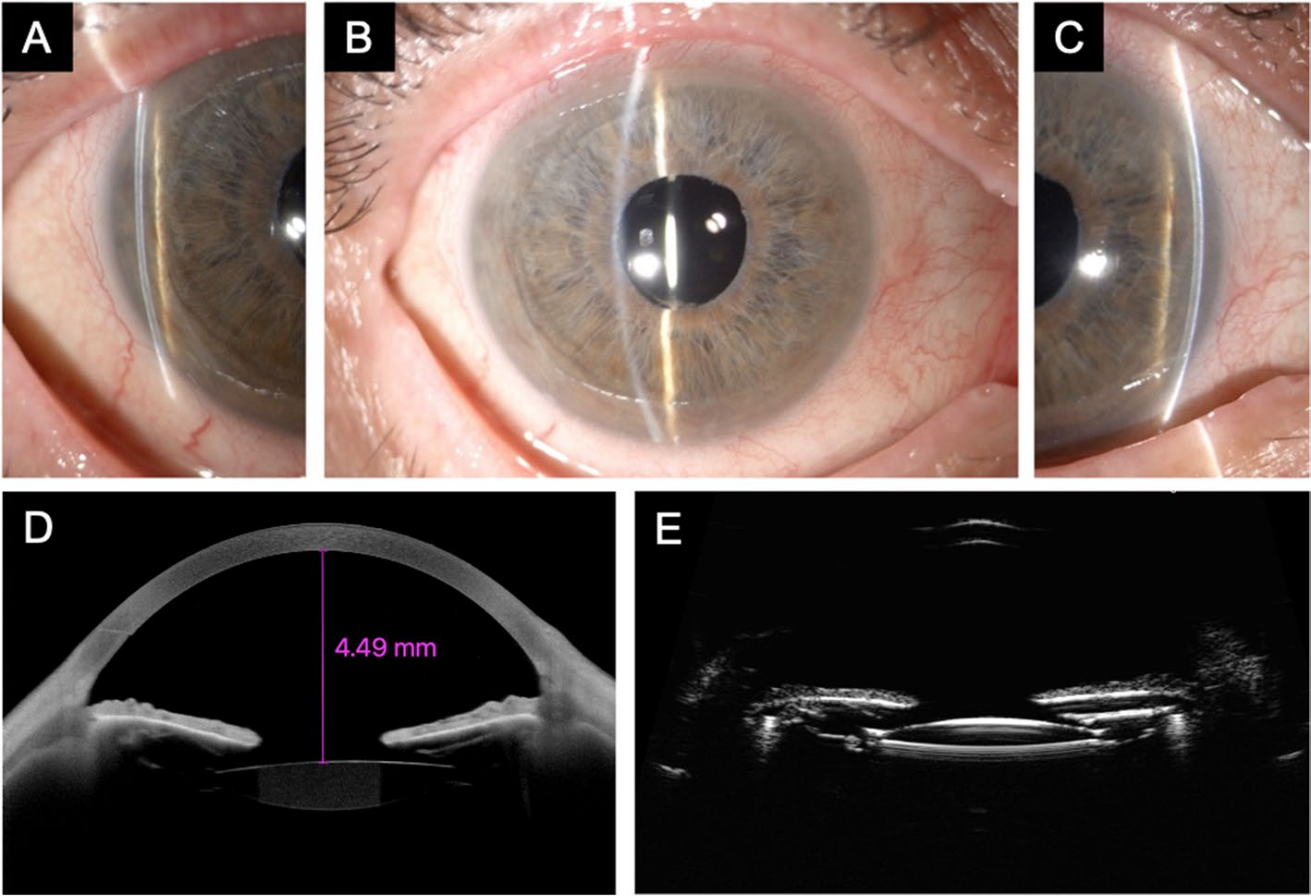
Anterior segment slit-lamp biomicroscopy of the same eye 12 months after pars plana anterior vitrectomy. The central and peripheral anterior chambers (**A**, **B**, and **C**) are deep (Van Herick grade 4). Anterior segment optical coherence tomography (**D**) and ultrasound biomicroscopy (**E**) show a deep anterior chamber and an open angle and a normal position of the iris-lens diaphragm. The untreated intraocular pressure was 12 mmHg

A single-port 25-gauge pars plana anterior vitrectomy was performed the following day without complications. On postoperative day 1, the IOP was 12 mmHg with no hypotensive medications. One month after surgery, the AC was deep, and the IOP was 10 mmHg without drugs. One year after surgery, the corrected visual acuity was 20/20 (Snellen), the IOP was 12 mmHg without antiglaucoma medications, the AC depth was 4.49 mm as measured at AS-OCT (Fig. 2A, B, and C), gonioscopy showed an open angle without irido-trabecular synechiae (Shaffer grade 4), and AS-OCT and UBM showed a normal AC configuration (Fig. 2D and E). No further complications were observed during the postoperative period.

## Discussion and conclusions

The term “malignant glaucoma” was first used by Albrecht von Graefe in 1869 to describe an aggressive form of postoperative glaucoma, also known as ciliary block or aqueous misdirection, characterised by shallowing of the AC with elevated IOP, resistance to treatment, and rapid evolution into blindness [14]. Diagnosis of malignant glaucoma includes a shallow central and peripheral AC associated with increased IOP despite a patent iridotomy and a normal posterior-segment anatomy. Although the etiology of this disease is not yet fully understood, the pathophysiology involves an anterior rotation of the ciliary body with misdirection of aqueous flow, resulting in forward shifting of the iris-lens diaphragm [15].

Malignant glaucoma has been reported following cataract surgery with or without intraocular lens implantation, as well as following glaucoma surgery, pars plana vitrectomy, laser capsulotomy, laser cyclophotocoagulation, laser iridotomy, laser scleral flap-suture lysis, trabeculectomy bleb needling, or in association with the use of topical miotic agents [16]. It represents a rare complication occurring more frequently after glaucoma filtering surgery, with a frequency of 0.4-6%, especially in eyes with angle-closure glaucoma [15]. Glaucoma filtration surgery is a risk factor for malignant glaucoma reported after drainage device implantation [17], iridencleisis (8.9%), and trabeculectomy (4.9%) [18].

MIGS have been proposed as safer and less traumatic surgical procedures characterised by an *ab interno* approach, minimal or no traumatic manipulation of the sclera and conjunctiva, a good safety profile, and rapid recovery. MIGS are known to have a lower complication rate than glaucoma filtration surgery, though they are not free from the risk of malignant glaucoma development [19]. Schlenker et al. reported four cases of malignant glaucoma after *ab interno* gelatin microstent (XENGelStent, Allergan, Inc., USA) implantation [20], and Montolío Marzo et al. reported a case of malignant glaucoma following phacoemulsification and XEN Gel Stent 45 implantation [21].

Since Grover et al. introduced GATT in the MIGS scenario, several encouraging results in terms of safety and efficacy have supported this novel technique. In their original description of the technique, Grover et al. [6] reported an IOP reduction from 24.7 to 15.7 mmHg at 12 months after surgery with a significant decrease in the number of medications in patients with POAG. In a longer-term study, IOP lowering results were nearly equivalent to the early ones [22]. The same study demonstrated greater GATT efficacy in patients with secondary open-angle glaucoma (OAG) with a decrease from 30.1 to 12.9 mmHg at 12 months.

Similarly, Rahmatnejad et al. [23], in a retrospective chart review of PAOG patients who underwent GATT, reported an average postoperative IOP of 14.6 mmHg with a 44% decrease at 12 months after surgery. Comparably, Aktas et al. [24] observed a 40.1% IOP reduction at 19 months in a retrospective study conducted in PAOG patients who underwent GATT using a 6-0 prolene suture.

Sharkawi et al. [9] recently demonstrated that GATT safely and effectively lowers the IOP in PEXG, either when performed alone or in combination with cataract surgery.

The most common postoperative complications described in large cohorts of patients treated with GATT are transient hyphema and IOP spikes [6, 8, 9, 23, 24]. Rarer complications include panscleritis in a patient with a history of anterior uveitis [25], postoperative intracapsular hematoma in a pseudophakic patient [26], and Descemet’s membrane detachment [27]. Recently, two cases of acute transient myopia secondary to supraciliary effusion without raised IOP have been described following GATT surgery [28].

To our knowledge, this is the first case of malignant glaucoma reported after an uneventful GATT procedure.

Although the exact pathogenesis of malignant glaucoma is not fully understood, different mechanisms have been proposed. Shaffer and Hoskins [29, 30] suggested misdirection of the aqueous humour backward into the vitreous cavity with secondary forward displacement of the iris-lens diaphragm. Chandler [31] proposed a laxity of lens zonules coupled with pressure from the vitreous humour, leading to forward lens movement, while Quigley et al. [32, 33] suggested choroidal expansion as a precipitating event that increases vitreous pressure [15]. In the case described herein, abnormal slackness and weakness of the lens zonules, commonly seen in PEXG, may have contributed to this complication.

Despite malignant glaucoma being generally infrequent after MIGS and never described after GATT, it is essential to recognise and treat this complication early to avoid permeant vision loss.

## Data Availability

All data generated and analysed during this study are included in this article.

## Abbreviations

GATT: Gonioscopy-assisted transluminal trabeculotomy
PEXG: Pseudoexfoliative glaucoma
AC: Anterior chamber
IOP: Intraocular pressure
POAG: Primary open-angle glaucoma
AS-OCT: Anterior segment optical coherence tomography
UBM: Ultrasound biomicroscopy
Nd:YAG: Neodymium-doped yttrium aluminium garnet
MIGS: Minimally invasive glaucoma surgery
